# Development and validation of a Greek language version of the Manchester Foot Pain and Disability Index

**DOI:** 10.1186/1477-7525-6-39

**Published:** 2008-06-01

**Authors:** Patricia Kaoulla, Nicoletta Frescos, Hylton B Menz

**Affiliations:** 1 Department of Podiatry, Faculty of Health Sciences, La Trobe University, Bundoora, Victoria 3086, Australia; 2 Musculoskeletal Research Centre, Faculty of Health Sciences, La Trobe University, Bundoora, Victoria 3086, Australia

## Abstract

**Background:**

The Manchester Foot Pain and Disability Index (MFPDI) is a 19 item questionnaire used to assess the severity and impact of foot pain. The aim of this study was to develop a Greek-language version of the MFPDI and to assess the instrument's psychometric properties.

**Methods:**

The MFPDI was translated into Greek by three bilingual content experts and two bilingual language experts, and then back-translated into English to assess for equivalence. The final Greek version was administered, along with a questionnaire consisting medical history and the Medical Outcomes Study Short Form 36 (SF-36), to 104 Greek-speaking, community-dwelling people (64 female, 40 male), aged between 64 and 90 years (mean 73.00, SD 5.26) with disabling foot pain.

**Results:**

The Greek translation of the MFPDI was found to have high internal consistency (Cronbach's α= 0.89, and item-total correlation coefficients from 0.33 to 0.72). Principal components analysis revealed a four-factor structure representing the constructs of functional limitation, pain intensity, concern with appearance and activity restriction, which explained 60.8% of the variance, with 38.9% of the variance explained by the first construct (functional limitation). Six items demonstrated different factor loadings to the original English version.

**Conclusion:**

The Greek-language version of the MFPDI appears to be a valid tool in assessing foot pain in Greek-speaking older people. The total MFPDI scores are comparable between the Greek and English version, however due to differences in the factor loadings of some items, between-language comparisons of MFPDI should be undertaken with some caution.

## Background

It has long been recognised that foot pain is common in older people and has a significant detrimental impact on mobility and quality of life. Community-based studies indicate that between 20 and 30% of community-dwelling people aged 65 years or over report foot pain [1-4]. Older people with foot pain demonstrate impaired balance and gait [3,5], report greater difficulty in performing activities of daily living [6], and have reduced health-related quality of life [7] compared to those without foot pain.

Several instruments have been developed to quantify the severity and impact of foot pain, including the American Orthopaedic Foot and Ankle Society scales [8], the Foot Function Index [9] and the Foot Health Status Questionnaire [10]. However, only one instrument – the Manchester Foot Pain and Disability Index (MFPDI) – has been validated in both middle-aged and older populations [11,12]. The MFPDI consists of 19 statements beginning with the phrase "Because of pain in my feet", which were initially found to cluster around three constructs: functional limitation (10 items), pain intensity (5 items) and concern with personal appearance (2 items) [11]. The remaining two items are related to the difficulty in performing work or leisure activities, which are excluded from the questionnaire if the respondent is of retirement age. A recent validation study in older people reported similar findings; however an additional fourth construct – activity restriction – was identified [12]. Since the initial development of the MFPDI, it has been applied in a population-based survey of foot pain [13] as an outcome measure in a clinical trial [14] and as a measure of foot pain in people with Ehlers-Danlos syndrome [15] and early rheumatoid arthritis [16].

The MFPDI appears to be a useful tool for the assessment of disabling foot pain in older people. However, the MFPDI has not been translated into other languages other than Swedish [15], thereby limiting the instrument's research potential. This is a particular problem in countries with large, ageing migrant populations, such as Australia, as many older migrants may not be sufficiently proficient in the English language to enable valid questionnaire data to be collected without the aid of a translator. Therefore, the aim of this study was to develop a Greek-language version of the MFPDI and to evaluate its internal consistency and construct validity.

## Methods

### Translation process

**Figure 1:**
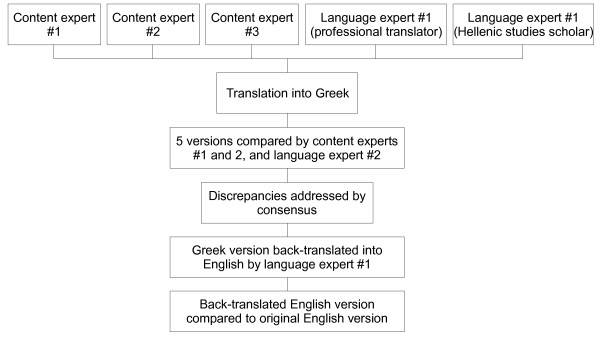
Translation process.

Three Greek-speaking content experts (two podiatrists and one foot and ankle surgeon) and two Greek-speaking linguistic experts independently translated the MFPDI into Greek. Three of the bilingual experts then evaluated the Greek versions, and any discrepancies were discussed and rectified by consensus. A professional Greek translator, who had no knowledge of the English questionnaire, independently back-translated the combined Greek language questionnaire into English. Three content experts, two Greek-speaking and the other non-Greek speaking, then compared the original English version to the back-translated questionnaire (see Figure 1).

### Participants

Participants in this study (n = 104) were recruited from four metropolitan elderly Greek citizen social groups in Melbourne, Australia. In order to recruit participants, a 10 minute presentation on foot disorders was delivered to each group. Included in the presentation was a brief outline of the study and a call for volunteers with foot pain to participate. The participants were required to be mobile and capable of walking household distances unaided, in order to evaluate the effect that foot pain has on routine mobility tasks. The study was approved by the Faculty of Health Sciences Human Ethics Committee of La Trobe University (application number: FHEC07/73) and informed consent was obtained from all participants.

### Medical history questionnaire and demographic information

A questionnaire relating to the participants' age, medical history and foot pain location was interviewer administered. The medical history section of the questionnaire required the participant to state whether they had any of the 15 common conditions listed. Participants who reported foot pain were asked to indicate the location of the pain on eight diagrams of the feet. The participants then completed the Greek language version of the MFPDI. A total score for the MFPDI was obtained according to the following scoring system for each of the 17 items: none of the time (score = 0), some days (score = 1), on most days/everyday (score = 2). General health-related quality of life was assessed with the validated Greek language version of the Medical Outcomes Study Short Form-36 (SF-36) [17].

### Foot assessment

Several clinical observations of foot structure were documented. Arch height was assessed by measuring the height of the navicular tuberosity in millimetres while the subject was fully weightbearing. This score was corrected for differences in foot size by dividing it by the length of the foot [18]. Ankle flexibility was measured in degrees using a modified version of the weightbearing lunge test. The lateral malleolus and head of the fibula was first located and marked with an ink pen. Participants then stood with their right foot placed alongside a vertically-aligned clear acrylic plate inscribed with 2° protractor markings, and were instructed to take a comfortable step forward with the left leg. In this position, participants were instructed to bend their knees to squat down as low as possible, without lifting the right heel from the ground and while keeping the trunk upright. The position of the fibular head was marked on the clear acrylic plate, and the angle formed between the lateral malleolus and the fibular head was measured. The test was completed three times, and the average score documented as the test result [19]. The presence and severity of hallux valgus ("bunions") was determined using the Manchester scale [20]. This instrument consists of standardised photographs of feet with four degrees of hallux valgus – none (score = 0), mild (score = 1), moderate (score = 2) and severe (score = 3) which were matched to the subject's feet. Gradings obtained using this scale are strongly associated with angular deformity measurements obtained from foot x-rays [21]. Presence of lesser digital deformity (hammertoes and clawtoes), corns and calluses were determined according to previously published criteria [22]. The reliability of these measurements performed on older people has been established previously [19].

### Statistical analysis

All statistical tests were conducted using SPSS Release 14 for Windows (SPSS Inc, Chicago, IL, USA). In order to determine the suitability of the data for principal components analysis, the Kaiser-Meyer-Olkin Measure of Sampling Adequacy (KMO) and Bartlett's Test of Sphericity were calculated. The KMO was found to be 0.84, which exceeds the recommended minimum value of 0.60 [23]. The Bartlett's Test of Sphericity was highly significant (χ^2 ^= 764, *p *< 0.001), supporting the suitability of the data for principal components analysis [24]. Internal consistency was determined using Cronbach's alpha and item-total correlation coefficients. A principal components analysis was then performed to determine whether the 17 items in the questionnaire could be combined into separate components reflecting different aspects of foot pain and disability. A four component solution was extracted using the Kaiser-Guttman rule (eigenvalues > 1.0), and varimax rotation was performed to minimize the complexity of loadings for each component. Correlations between the total MFPDI score with the SF-36 subscales were determined using the Pearson's *r *correlation coefficient.

## Results

### Translational issues

**Figure 2:**
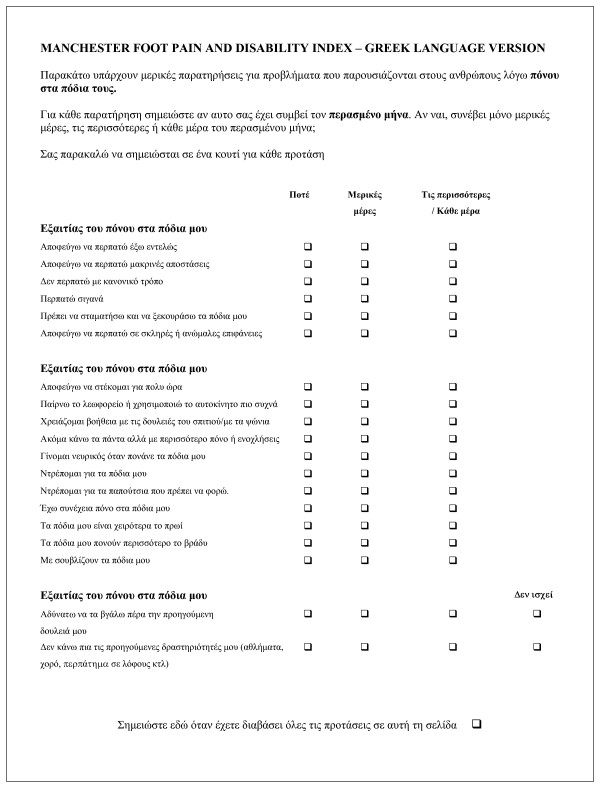
Greek language version of the Manchester Foot Pain and Disability Index.

Differences between the original English version and the back-translated version of the Manchester Foot Pain and Disability Index can be seen in Table 1. Six of the seventeen items used in this study were back translated identically to the original questionnaire. There were difficulties in translating two of the seventeen items: "My feet are more painful in the evening" (item 16) and "I get shooting pains in my feet" (item 17). The final Greek version is shown in Figure 2.

**Table 1:** Original MFPDI questions compared to the back translated MFPDI questions.

*Original MFPDI questions*	*Back-translated MFPDI questions*
I avoid walking outside at all	I totally avoid walking outside
I avoid walking long distances	I avoid walking long distances*
I don't walk in a normal way	I don't walk in a normal way*
I walk slowly	I walk slowly*
I have to stop and rest my feet	I need to stop and rest my feet
I avoid hard or rough surfaces when possible	I avoid walking on hard or uneven surfaces
I avoid standing for a long time	I avoid standing for long periods
I catch the bus or use the car more often	I take the bus or use the car more often
I need help with housework/shopping	I require help with jobs around the home/with shopping
I still do everything but with more pain or discomfort	I still do everything but with more pain or discomfort *
I get irritable when my feet hurt	I become irritable when my feet ache
I feel self-conscious about my feet	I am embarrassed of my feet
I get self-conscious about the shoes I have to wear	I am embarrassed of the shoes I have to wear
I have constant pain in my feet	I have constant pain in my feet*
My feet are worse in the morning	My feet are worse in the morning*
My feet are more painful in the evening	My feet are more painful at night
I get shooting pains in my feet	I have stabbing pains in my feet
I am unable to carry out my previous work	I am unable to cope with my previous job†
I no longer do all my previous activities (sport, dancing, hill-walking, etc)	I no longer do my former activities (sport, dancing, hiking etc)†

NB: * word-perfect back-translation, † Questions excluded from this study, as participants were of retirement age

### Characteristics of sample

The characteristics of the sample, including sex, prevalence of major medical conditions and medication use is shown in Table 2. The average age of the participants was 73 years (SD 5.3). The most common medical conditions amongst the participants included heart problems (61.5%), peripheral vascular disease (57.7%) and osteoarthritis (76.9%). More than forty per cent of the participants (41.3%) were taking four or more medications.

**Table 2:** Sample characteristics.

Age (years) – mean (SD)	73.0 (5.3)
Sex – n (%)	
Female	64 (61.5)
Male	40 (38.5)
	
Country of birth – n (%)	
Greece	59 (56.7)
Cyprus	41 (39.4)
Egypt	2 (1.9)
Armenia	1 (1.0)
Australia	1 (1.0)
	
Education: total years – median (range)	5.5 (0 – 17)
	
Major medical conditions – n (%)	
Heart problems	64 (61.5)
Peripheral vascular disease	60 (57.7)
Osteoarthritis	80 (76.9)
Hips	57 (54.8)
Hands/wrists	41 (39.4)
Spine	39 (37.5)
Knees	24 (23.1)
Feet	9 (8.7)
Cancer	24 (23.1)
Stroke	10 (9.6)
Diabetes	5 (4.8)
Rheumatoid arthritis	5 (4.8)
	
More than four medications – n (%)	43 (41.3)
	
Podiatry-related issues – n (%)	
Currently receives podiatry	13 (12.5)
Previous foot surgery	6 (5.8)
Difficulty finding comfortable shoes	39 (37.5)
Needs more help looking after feet	42 (40.4)
	
Foot characteristics	
Moderate to severe hallux valgus – n (%)	18 (17.0)
Calluses	29 (28.8)
Ankle flexibility (°)	40.1 (12.2)
Navicular height/foot length (mm)	0.11 (0.03)

### Internal consistency

The Cronbach's α calculation for the 17 items of the MFPDI was 0.89, indicating a high degree of internal consistency. The item-total correlation coefficients were generally between 0.45 and 0.72, with two exceptions: item 13 (0.37) and item 16 (0.33).

### Floor and ceiling effects

Frequencies of participants' responses to individual items of the MFPDI are shown in Table 3. All items exhibited a good spread of responses across the three categories, with no item demonstrating clear floor or ceiling effects. The items with the highest proportion of "on most days/every day" responses were "I avoid standing for a long time" (56%) and "I avoid walking distances" (46%), whereas the items with the lowest proportion were "I feel self-conscious about my feet" (10%) and "I get self-conscious about the shoes I have to wear" (6%).

**Table 3:** Frequencies of responses to individual items on the Greek MFPDI. Data shown as n (%).

Item	None of the time	On some days	On most days/every day
1. I avoid walking outside at all	69 (66)	20 (19)	15 (14)
2. I avoid walking distances	32 (31)	24 (23)	48 (46)
3. I don't walk in a normal way	52 (50)	29 (28)	23 (22)
4. I walk slowly	36 (35)	30 (29)	38 (36)
5. I have to stop and rest my feet	44 (42)	21 (20)	39 (38)
6. I avoid hard or rough surfaces where possible	35 (34)	29 (28)	40 (39)
7. I avoid standing for a long time	22 (21)	24 (23)	58 (56)
8. I catch the bus or use the car more often	24 (23)	46 (44)	34 (33)
9. I need help with housework/shopping	65 (63)	23 (22)	16 (15)
10. I still do everything but with more pain or discomfort	25 (24)	39 (38)	40 (39)
11. I get irritable when my feet hurt	40 (39)	42 (40)	22 (21)
12. I feel self-conscious about my feet	74 (71)	20 (19)	10 (10)
13. I get self-conscious about the shoes I have to wear	92 (89)	6 (6)	6 (6)
14. I have constant pain in feet	36 (35)	35 (34)	33 (32)
15. My feet are worse in the morning	61 (59)	14 (14)	29 (28)
16. My feet are more painful in the evening	53 (51)	30 (29)	21 (20)
17. I get shooting pains in my feet	41 (39)	36 (35)	27 (26)

### Principal components analysis

Results of the principal components analysis are shown in Table 4, along with the factor structure reported in the original validation study of English-speaking older people [12]. A four-factor model was extracted which accounted for 61% of the total variance. However, the majority of the variance was explained by the first component (38.9%).

**Table 4:** Component coefficients of the individual items of the MFPDI in older people and comparison to factor structure of Menz et al [12].

	Component 1	Component 2	Component 3	Component 4	Factor structure	
		
% variance explained	38.9	8.2	7.1	6.6	Menz et al [12].	Current study
	
Item						
1. I avoid walking outside at all	**0.592**	0.136	-0.423	0.386	AR	FL*
2. I avoid walking distances	**0.702**	-0.029	-0.429	-0.042	FL	FL
3. I don't walk in a normal way	**0.703**	-0.122	0.147	0.121	FL	FL
4. I walk slowly	**0.603**	-0.473	-0.150	0.051	FL	FL
5. I have to stop and rest my feet	**0.773**	-0.182	0.072	-0.061	FL	FL
6. I avoid hard or rough surfaces where possible	**0.641**	-0.084	-0.283	-0.283	FL	FL
7. I avoid standing for a long time	**0.636**	-0.289	-0.063	0.116	FL	FL
8. I catch the bus or use the car more often	**0.539**	0.218	-0.151	**0.527**	FL	FL/AR*
9. I need help with housework/shopping	**0.644**	-0.145	0.077	-0.206	AR	FL
10. I still do everything but with more pain or discomfort	**0.707**	0.033	0.013	-0.377	PI	FL*
11. I get irritable when my feet hurt	**0.650**	0.303	-0.044	0.025	PI	FL*
12. I feel self-conscious about my feet	**0.551**	0.278	0.470	-0.263	CA	FL*
13. I get self-conscious about the shoes I have to wear	0.420	0.101	**0.599**	0.470	CA	CA
14. I have constant pain in feet	**0.768**	0.178	0.134	-0.113	PI	FL*
15. My feet are worse in the morning	0.483	**0.601**	0.285	0.161	PI	PI
16. My feet are more painful in the evening	0.392	**0.522**	-0.078	0.107	PI	PI
17. I get shooting pains in my feet	**0.643**	0.309	0.055	-0.226	PI	FL*

Notes: FL – functional limitation, PI – pain intensity, CA – concern about appearance, AR – activity restriction. Factor loadings > 0.45 highlighted in bold. *Different factor loading

Component 1 represented 13 items pertaining to functional limitation (items 1–7, 9–12, 14, 17), component 2 represented two items pertaining to pain intensity (items 15 and 16), component 3 represented one item pertaining to concern about appearance (item 13), and component 4 represented one item pertaining to activity restriction (item 8, which also loaded onto component 1).

### Correlates of total MFPDI score

Women had a higher total MFPDI score than men (15.64 *vs*. 11.63; *t *= -2.46, *df *= 102, *p *= 0.01). The total MFPDI score was significantly associated with each of the subscales of the SF-36: physical (*r *= -0.66, *p *< 0.001), role-physical (*r *= -0.56, *p *< 0.001), bodily pain (*r *= -0.66, *p *< 0.001), general health (*r *= -0.56, *p *< 0.001), vitality (*r *= -0.68, *p *< 0.001), social function (*r *= -0.53, *p *< 0.001), role-emotional (*r *= -0.49, *p *< 0.001) and mental health (*r *= -0.53, *p *< 0.001). There were no significant associations between any of the foot assessment variables and MFPDI scores.

## Discussion

This study was undertaken to develop a Greek-language version of the Manchester Foot Pain and Disability Index and to assess its psychometric properties. Compared to other languages, Greek does not have any significant variations in the use of words, however some regional differences exist. The Greek questionnaire developed in this study used "standard" Greek and avoided colloquialisms in order to allow all Greek-speaking people to understand it. This was important in avoiding differences in the psychometric properties of the questionnaire.

The translation process was relatively straightforward. Six of the seventeen items (items 2, 3, 4, 10, 14, and 15) were back-translated identically to the original questionnaire. The remaining nine items (item 1, 5, 6, 7, 8, 9, 11, 12, 13) were very similarly back translated to the original questionnaire. However, there were two significant translational issues. Firstly, item 16, "My feet are more painful in the evening" was back-translated into "My feet are more painful at night"; however the Greek word for "night" (νύχτα) is different from the word used for "evening" (βράδυ). To be consistent with the original MFPDI, "evening" was retained. The most difficult translational issue was with item 17, "I get shooting pains in my feet". The Greek word for "shooting" (τουφεκισμόζ) is a literal translation for the act of shooting a gun; however, this word is clearly not appropriate for describing pain. After lengthy discussions regarding this item, an alternative word meaning "piercing" (σουβλίζουν) was decided on by the three bilingual content experts. This item was back-translated to "I have *stabbing *pains in my feet". Although there was difficulty in translating item 17, most of the participants (60.6%) claimed that they experienced this disability on some (34.6%) or most/every day (26%), thus suggesting that it was understood as a descriptor of pain in this population.

The psychometric properties of the Greek MFPDI were similar to the original English validation studies in middle-aged [11] and older participants [12]. Internal consistency reported in our study was the same as that reported by Menz et al. [12] but slightly lower than that reported by Garrow et al. [11] in the initial validation study (Cronbach's α = 0.89 compared to 0.99). A Cronbach's α of 0.89 may be more desirable, as it is well above the acceptable limit of 0.70 [25]. A very high Cronbach's α, such as that reported by Garrow et al. [11] may indicate some degree of redundancy in the MFPDI when assessing foot pain in middle-aged people.

Similar to the initial validation study [11], the principal components analysis revealed a four-component solution, strongly reflecting the three constructs of functional limitation, pain intensity and concern with personal appearance. These results differ from the study that validated the MFPDI in English-speaking older people that found an additional construct relating to activity restriction, describing a more severe impairment than functional limitation [12]. In the current study we only found a weak loading for this fourth construct. Furthermore, item 8 ("I catch the bus or use the car more often") exhibited some degree of cross-loading, in that two reasonably high component coefficients (>0.5) were split across two components (1 and 4). However, this is not unique to the Greek version, as the original MFPDI exhibited cross-loading on several items [11]. Although some authors have suggested that cross-loading items should be deleted [26], we decided to retain this item in order for the instrument to be as similar as possible to the original MFPDI.

Similar to the original validation study, the two items "I avoid walking outside at all" and "I need help with housework/shopping" were found to relate to functional limitation, which differs to the finding of Menz et al. [12] who reported these disabilities to be associated with a distinct fourth construct – activity restriction. This indicates that older Greek-speaking people may perceive these items as less severe symptoms than English-speaking older people, i.e.: they consider these statements as having a reduced ability to perform the tasks, not an inability to complete them.

Another difference in the factor loading between this study and that of Menz et al. [12] was that item 11 ("I get irritable when my feet hurt") was located in the functional limitation component. As with Garrow et al. [11], our results suggest that Greek older people seem to relate to this item as the frustration associated to the impaired functional ability due to foot pain, rather than as a measure of the sensory experience of pain. Based on the different constructs revealed by principal components analysis, comparison of the subscales may not be possible when comparing the results of the Greek language version MFPDI to the original English version, however further study in a more representative sample would be necessary to confirm this. Nevertheless, comparison of the total scores may be a useful measure in determining and comparing the severity of disabling foot pain in Greek and English-speaking older people.

Construct validity was evidenced by significant correlations between the Greek language MFPDI with all the subscales of the previously validated Greek language version of the SF-36 questionnaire [17]. The MFPDI and SF-36 purport to measure foot-specific and generic health-related quality of life, respectively, so it was expected that older people who scored poorly on one scale would also score poorly on the other. However, there is a possibility that these high correlations partly reflect some degree of misinterpretation on behalf of the participants when completing the MFPDI. As with the English version, the Greek translation has the prefix "Because of pain in my feet..." before each of the items, however previous applications of the English version by Garrow et al. [11] and Menz and Morris [19] have noted that some participants respond to the MFPDI questions from the perspective of their general health, and need to be reminded that the questions pertain specifically to their feet.

Concurrent validity of the translated scale was evidenced by significant differences in the total MFPDI scores of male and female participants. This is consistent with previous observations that women are more likely to report foot pain [3,4,27], due to an increased prevalence of hallux valgus, corns and calluses [1,2,28], the wearing of ill-fitting footwear [29], and sex differences in pain tolerance levels [30].

One limitation of this study is that due to time constraints, the participants were conveniently recruited from Greek elderly citizen groups. As such, they were all independent community dwelling older people, capable of walking household distances. The participants all reported some degree of foot pain, however many of them had never received foot care. It is therefore unclear as to whether the findings may be generalisable to people outside this group, such as less mobile older people with more severe pathologies requiring treatment from foot care specialists.

## Conclusion

The findings of this study indicate that the Greek language version of the Manchester Foot Pain and Disability Index is a useful tool in assessing the severity of disabling foot pain in Greek-speaking older people, although the component structure in our sample differs from the validation of the instrument in English-speaking older people. Increased use of this instrument in both epidemiological studies and clinical trials will further develop our understanding of foot pain in the Greek-speaking population.

## Abbreviations

MFPDI: Manchester Foot Pain and Disability Index; SF-36: Medical Outcomes Study Short Form 36.

## Competing interests

The authors declare that they have no competing interests.

## Authors' contributions

NF and HBM conceived the study design, HBM conducted the statistical analysis, PK collected the data, and all authors interpreted the results, drafted the manuscript, and read and approved the final manuscript.
